# Factors Influencing Delay in Seeking Care for Mental Illness Among a Sample of Adult Saudi Arabian Patients

**DOI:** 10.7759/cureus.49438

**Published:** 2023-11-26

**Authors:** Yasir Altuwairqi

**Affiliations:** 1 Medicine/Psychiatry, Taif University, Ta'if, SAU

**Keywords:** depression, anxiety, psychosis, delay, psychiatric illness

## Abstract

Background: Treatment and prognosis of psychiatric disorders could be negatively affected by delay in seeking mental health care. The development of mental health services depends on understanding the reasons for delays in help-seeking and pathways to care and the duration of illness before treatment is initiated.

Objectives: The aim of the present study is to examine the reasons why patients with psychiatric symptoms delay their visits to psychiatry clinics in Saudi Arabia.

Methods: This was a cross-sectional, observational, survey-based study that included 268 patients, of which 60.8% were males. Data were collected through a questionnaire (either online or distributed to patients who attend the outpatient psychiatric clinics) in Taif, Saudi Arabia.

Results: Most patients were married males below 40 years old, with a university degree level, employed, and having average financial status. The most common symptom reported by responders was anxiety (41.8%). The most common reason in both age groups was the fear of side effects of medication (31.9% in those over 40 years and 18.4% below 40 years), followed by the patient belief that these are non-psychiatric symptoms and will disappear with time. The trial of folk medicine was the least common reason for both age groups (1.8% in patients below 40 years and 0% for patients above 40 years).

Conclusion: Fear of psychotropic drug adverse effects was the most frequent reason given for postponing mental health counseling. This could be due to some unpleasant or intolerable effects. Anxiety was the most common psychiatric symptom among patients delaying their first psychiatric consultation. These findings serve as a guide for the improvement of mental health services and psychoeducation in Saudi Arabia.

## Introduction

The World Health Organization (WHO) ranked Saudi Arabia 26th out of 179 countries in providing the best healthcare for their populations in general, not just mental healthcare [1].

Statistical data for the mentally ill in Saudi Arabia shows that the number of mentally ill patients has increased rapidly from 10,245 patients in inpatient units in 2002 to 15,004 in 2004 [2]. More recent data showed that the prevalence of a mental disorder among Saudi youth was 40.10%, where anxiety disorders affected 26.84% of the sample, followed by disruptive behavior disorders (15.44%), mood disorders (9.67%), substance use disorders (4%), and eating disorders (7.06%) [3]. The prevalence of mental disorders among patients who attended primary healthcare centers was 28.5% [4].

The Saudi Ministry of Health (MOH), as part of its mental health strategy for Vision 2030, has recently initiated a new healthcare system to help people physically, socially, and mentally through a new patient-centered model of care. A “National Committee for Mental Health Promotion” has been established with cross-sectorial stakeholder collaboration and involvement, ensuring it is aligned with the integration of services starting from the community level [5]. To develop mental health services, it is crucial to comprehend how people seek medical attention for illness. Understanding the pathways to care, barriers to help-seeking, and the time between illness onset and treatment initiation is essential.

Studies showed many factors behind the delay in seeking psychiatric help. Those factors were (a) financial strain required by mental health services [6] and uneasy access to the healthcare provider due to transportation or insufficient resources [7-8]; (b) personal beliefs, such as lack of awareness of the need for treatment [9] and doubt about the efficacy of the therapy [10-11]; and (c) stigma. Cultural factors could also influence intentions to seek help [12]. A study carried out in Malaysia endorsed the above results and showed that self-stigma and early ages correlated negatively with psychiatric help-seeking attitudes among students from low-income households [13].

The results of these studies certainly help us understand the reasons for the delay in seeking psychiatric help. However, these studies come from different cultures (Western countries and Malaysia), so caution should be exercised when interpreting their results.

Studies conducted in Saudi Arabia found that poor knowledge and negative attitudes were reported by both non-psychiatric doctors and patients, which negatively influenced the referral rates [14-15]. These two studies primarily focused on the phenomenon of stigma, particularly within the context of in-patient settings. In contrast, our study offers a more comprehensive examination of the various factors contributing to the delay in seeking healthcare, rather than being specific to a particular in-patient setting. The stigma of mental illness was the most reported barrier that prevented Saudi patients from seeking mental healthcare [16]. Many reasons for the stigma of mental illness were reported (e.g., cultural norms, belief that psychiatric disorders cannot be cured, and lack of community awareness about the role of psychiatric services) [17]. Delay in seeking medical care often results in more complications and poor outcomes. In addition to psychotic patients, delaying help-seeking for anxiety and depression patients can lead to prolonged suffering, worsening of symptoms, functional impairment, and a higher risk of negative outcomes [18-19].

The literature review revealed the scarcity of data about the reasons for the delay in seeking mental healthcare in Saudi Arabia. Therefore, we aimed to investigate the factors causing a delay in seeking mental healthcare in Saudi Arabia.

## Materials and methods

Study design

A cross-sectional, observational design was conducted in this study. A questionnaire was designed by the researcher and distributed manually to patients attending psychiatric clinics in Taif Mental Hospital (107), Taif, Saudi Arabia. As an additional data collection method, an online data collection form similar to the manual one was designed and administered through Google Forms (161).

Study population

A total of 268 patients had psychiatric symptoms during the period from 6 January 2019 to 7 February 2019. The sample size was calculated considering a marginal error of 5% and a confidence level of 90% and considering the maximum uncertainty. These assumptions gave a minimum sample size of 267 participants. We followed a consecutive sampling technique, and all eligible patients attending psychiatric clinics in Taif Mental Hospital during the data collection period were consecutively invited to take part in the study.

Data collection

The questionnaire contained three main sections covering the study endpoints with closed-ended questions.

The first section included demographic data: age, gender, level of education, career, nationality, and financial level.

The second section included questions about the mental suffering of patients comprising the main psychiatric diagnostic categories, namely, psychosis, depression, anxiety, addiction, and other diagnoses. The time interval between the onset of the last psychiatric symptoms and the first visit to a psychiatrist was included. Based on the interval between the onset of symptoms and the psychiatrist visit, the following categories were used: 0-1 month, one to three months, three to six months, 6-12 months, one to two years, and more than two years.

The last section included questions about the common reasons for the delay in seeking psychiatric help after the onset of symptoms. This section of the questionnaire was developed by the author based on his clinical experience with the target population over 20 years and his familiarity with local culture and customs.

Inclusion/exclusion criteria

Psychiatric patients aged between 18 and 65 years old of both genders were included, while patients with neurocognitive deficits (forgetfulness or inability to comprehend the questionnaire) were excluded.

Statistical analysis

All statistical calculations were performed using IBM SPSS (Statistical Product and Service Solutions; IBM Corp, Armonk, NY), version 21, for Microsoft Windows. An Excel sheet was used to enter the data collected from the survey. Data were represented in the form of frequencies (number of responders) and valid percentages for categorical variables. A chi-square test was used to compare different subgroups. All P values < 0.05 were considered statistically significant.

## Results

The present study surveyed 268 patients with psychiatric symptoms, some of whom have presented to the psychiatric clinic.

Descriptive analysis

Demographic Data

Of the 268 participants, 163 (60.8%) were males, and 105 (39.2%) were females. The age of patients was categorized into two age groups, either below 40 years old or above 40 years old. Here, 81.6% of the participants were from the age group less than 40 years old. Moreover, 42.3% did not have a job. As for education, 54.5% of the cohort had a university degree, while 86.3% lived in urbanized areas. The financial status of 56% of the participants was described as average. Table 1 represents all the demographic data. 

**Table 1:** Demographic data of the participants

Parameter	Frequency	Percent
Gender		
Males	163	60.8
Females	105	39.2
Age (in years)		
Less than 40 years old	218	81.6
More than 40 years old	49	18.4
Employment		
Employed	106	39.7
Unemployed	113	42.3
Student	48	18.0
Education		
Primary	15	5.6
Intermediate	21	7.8
Secondary	73	27.2
University degree	146	54.5
Illiterate	13	4.9
Residence		
Urban	232	86.9
Rural	35	13.1
Marital status		
Single	125	46.6
Married	130	48.5
Divorced	12	4.5
Widowed	1	0.4
Financial status		
Low	48	17.9
Average	150	56.0
Good	70	26.1
Response type		
OPD patients (online data)	161	60.1
Community patients (paper data)	107	39.9

Description of psychiatric symptoms

**Figure 1:**
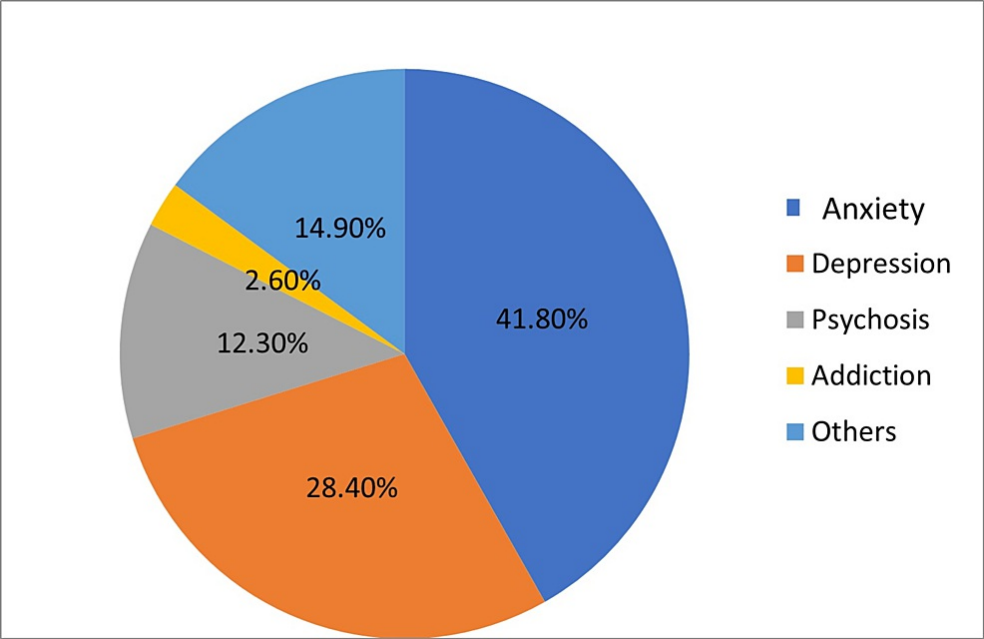
Distribution of psychiatric symptoms in percent Other symptoms included eating disorders, sleep disturbance, behavioral disorders, etc.

**Figure 2:**
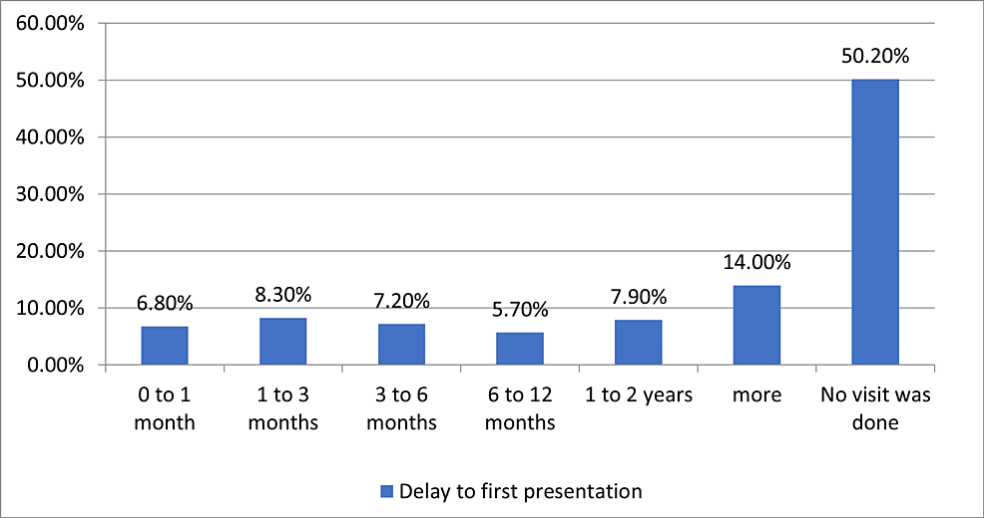
Delay in first psychiatric presentation

Section two of the survey involved questions about psychiatric symptoms. The most common symptom was anxiety, as reported by 41.8% of the participants. On the other hand, addiction was the least reported symptom (2.6%). Additionally, most patients had symptoms for more than two years (36.2%). Additionally, 34.8% of the participants reported that the delay between the onset of symptoms, and their first visit to the psychiatrist was more than three months. Patients were asked about their family history for any psychiatric symptoms, and 64.1% of the participants did not have any positive family history. The distribution of psychiatric symptoms is shown in Figure 1, while Figure 2 shows the delayed duration of the first psychiatric consultation.

In the questionnaire, patients were asked about the reasons for their delay in their first psychiatric consultation. The most common reason appeared to be "fears from the adverse effects of psychiatric medications" (20.8%), while the least common reason was "going to folk medicine practitioners or “Sheikh” to resolve their symptoms" (1.5%). A description of all the reasons is shown in Table 2.

**Table 2:** Reasons for delays to the first psychiatric presentation

Reasons	Frequency	Percent
Fear of psychiatric adverse events of medications	55	20.8
Feeling embarrassed from psychiatric symptoms	24	9.1
Unavailability of psychiatric treatment	8	3.0
Un-realizing the need for psychiatric treatment	23	8.7
Symptoms were not clear	27	10.2
Believing that these are non-psychiatric symptoms	34	12.8
Going to a Sheikh or folk medicine practitioner	4	1.5
Believing that symptoms will disappear with time	29	10.9
Absence of anyone to help the patient or take him to a psychiatrist	10	3.8
Refusing to go to a psychiatrist	8	3.0
Financial reasons	7	2.6
Others (e.g., self-treatment, no nearby facility)	35	13.2

The age groups of the participants were correlated to many factors, such as psychiatric symptoms, symptoms duration, and delay to the first visit using the chi-square test at a level of significance p value < 0.05. All details are provided in Table 3.

**Table 3:** Correlation between age groups and psychiatric symptoms, psychiatric symptoms duration, delay to the first presentation, and type of the first visit *Level of significance at P value < 0.05

Patient data	Patient sub-data	Less than 40 years	More than 40 Years	P value
Psychiatric symptoms	Anxiety	39.9%	51.0%	0.016*
	Depression	32.6%	10.2%	
	psychosis	11.9%	12.2%	
	addiction	1.8%	6.1%	
	others	13.8%	20.4%	
Psychiatric symptoms duration	0-1 month	16.1%	22.4%	0.320
	1-3 months	10.6%	6.1%	
	3-6 months	11.9%	4.1%	
	6-12 months	9.2%	8.2%	
	1-2 years	18.3%	14.3%	
	More	33.9%	44.9%	
Duration of delay to first psychiatrist visit	0-1 month	7.0%	6.1%	0.917
	1-3 months	7.9%	10.2%	
	3-6 months	7.0%	8.2%	
	6-12 months	6.0%	4.1%	
	1-2 years	8.4%	6.1%	
	More	12.6%	18.4%	
	No visit was done	51.2%	46.9%	
The first visit was to	Sheikh	36.4%	26.1%	0.715
	Folk medicine or non-psychiatrist	3.7%	4.3%	
	Physician (not a psychiatrist)	10.3%	13.0%	
	No visit	49.1%	56.5%	

Additionally, both age groups were correlated to the reason for delaying the first psychiatrist visit. Although the difference in reasons between both age groups was statistically non-significant (p = 0.124), it was revealed that fear of the adverse effects of psychiatric medications was the most common reason for both age groups. Almost a third (31.9%) of the patients above 40 feared adverse effects of medications compared to (18.4%) in the age group below 40. It is also worth mentioning that none of the participants above 40 tried folk medicine, and none of them refused to go to a psychiatrist, compared to 1.8% and 3.7%, respectively, in the age group below 40. Additionally, trying folk medicine was the minor reason for the delay in the first psychiatrist visit.

For the same age groups, no statistically significant difference was observed upon correlating patients' gender to the reasons for delaying the first psychiatric visits (p = 0.064). However, the most prevalent reason among males was the fear of psychiatric adverse events of medications (25.6%), while for females the most frequent reason was believing that the symptoms would disappear over time (15.2%).

Moreover, the reasons for delay were compared between patients with anxiety and those with depression. Although no statistically significant difference was revealed (p = 0.450), a larger proportion of patients with anxiety reported feeling embraced from psychiatric symptoms (10.7%) and not realizing the need for psychiatric treatment (8.0%) as the most frequent reasons for delay compared to patients with anxiety (5.4% and 3.6%).

More details are provided in Table 4.

**Table 4:** Reasons for delays to the first psychiatrist consultations in the different subgroups

	Age group (p=0.124)		Gender (p=0.064)		Psychiatric symptoms (p=0.450)	
Reasons	Less than 40 years	More than 40 Years	Male	Female	Anxiety	Depression
Fear of psychiatric adverse events of medications	18.4%	31.9%	25.6%	13.3%	24.1%	25.3%
Feeling embraced from psychiatric symptoms	9.2%	8.5%	7.5%	11.4%	5.4%	10.7%
Unavailability of psychiatric treatment	2.8%	4.3%	3.8%	1.9%	4.5%	2.7%
Un-realizing the need for psychiatric treatment	8.3%	10.6%	10.6%	5.7%	3.6%	8.0%
Symptoms were not clear	11.1%	6.4%	11.3%	8.6%	11.6%	5.3%
Believing that these are non-psychiatric symptoms	14.3%	6.4%	11.9%	14.3%	16.1%	12.0%
Going to Sheikh or a folk medicine practitioner	1.8%	0.0%	1.9%	1.0%	1.8%	2.7%
Believing that symptoms will disappear over time	8.8%	21.3%	8.1%	15.2%	12.5%	13.3%
Absence of anyone to help the patient take him to a psychiatrist	4.1%	2.1%	2.5%	5.7%	2.7%	4.0%
Refusing to go to a psychiatrist	3.7%	0.0%	3.8%	1.9%	0.9%	4.0%
Financial reasons	3.2%	0.0%	3.1%	1.9%	4.5%	1.3%
Others (e.g., self-treatment, no nearby facility)	13.8%	8.5%	9.4%	19.0%	12.5%	9.3%

## Discussion

The present work aimed to explore various reasons that could make psychiatric patients in Saudi Arabia delay their first visit to a psychiatrist when they have symptoms of psychiatric illness. Most patients were married males below 40 years old, with a university degree, employed, and with an average financial status. The most common symptom reported by responders was anxiety (41.8%). Furthermore, most responders (34.8%) delayed their first visit to a psychiatrist for more than three months, and 50% of patients did not seek psychiatric help at all. This can be explained positively that their symptoms were mild. Another negative explanation is that patients have poor psychoeducation to present to a psychiatric facility at the proper time or lack awareness of their psychiatric symptoms.

Many psychiatric patients present with severe symptoms and advanced stages of the disease on their first visit to the psychiatrist. This could be explained by the delayed visit of patients for multiple reasons. These reasons include awareness of symptoms and the need for psychiatric help. Birchwood et al. [20] studied the duration of untreated psychosis; this study yields that a longer duration of untreated illness is associated with poor outcomes, including symptom exacerbations and neurological complications [20-21]. Moreover, it is hypothesized that a period of five years is required from the psychosis onset to establish intervention [22]. The above results were endorsed by other studies, which indicated that early intervention could decrease morbidity and the cost of illness [23,24]. Additionally, the delay in help-seeking for anxiety and depression patients can lead to more complications, chronic course, and poor outcomes [18,19]. Addressing the factors influencing the delay in the first psychiatrist visit is crucial to reducing undesirable delays [25]. Therefore, efforts should be made to explore the factors influencing the delay in the first visit to psychiatric clinics. In our study, nearly half of the participants who attended psychiatric clinics reported being delayed for more than one to two years. This can lead to more chronic and complicated diseases, leading to a greater burden on patients and increased utilization of healthcare resources. These results agreed with Norman et al. where the duration of untreated psychosis was approximately one to two years [26].

Turning to the reasons for delaying the visit to a psychiatrist, the most common reason in both age groups was the fear of the side effects of medication (31.9% in those more than 40 years and 18.4% below 40 years). This emphasizes how crucial psychoeducation is regarding the adverse effects of psychiatric medications. Educating our patients about the safety and tolerability of psychotropic medication and the fact that almost all adverse effects are not serious will help them accept taking their medication and improve their conditions. Consequently, they will be in better shape and society will benefit as well. On the other hand, trying folk medicine was the least common reason for both age groups (1.8% for patients below 40 years and 0% for patients above 40 years).

Similarly, Altamura et al. [27] evaluated the factors influencing the duration of the first psychiatric consultation; however, their study focused on patients with bipolar disorder in Italy only. This study included 320 patients in an interview to ask them about their symptoms and followed them up for five years to explore the clinical features of the disease and how symptoms differed before and after treatment. It also showed that factors associated with the longer duration of untreated illness were a higher incidence of suicidal attempts and aging. Moreover, it confirmed a correlation between the worsened outcome of bipolar disease and the prolonged duration of untreated illness. These results comply with the findings of the present study despite the difference in the populations recruited. However, the present study included fewer patients and did not follow up with patients before and after treatment.

On the other hand, the present study included disorders other than bipolar disease, which gives a better description of more common diseases. Moreover, the present work described the correlation between factors influencing delayed untreated illness and the age of the patients, revealing that fear of side effects of medication was the most common reason for delaying the first psychiatric visit (31.9% in those more than 40 years and 18.4% below 40 years).

On the subject of psychiatric symptoms, there was a significant difference (p = 0.016) between symptoms in patients below and above 40 years old. Anxiety came in the first rank, with 39.9% of patients less than 40 years old and 51.0% of patients above 40 having anxiety. Similar findings were also supported by Callaghan et al. [28] and Weiss et al. [29] in different healthcare settings and different populations.

Unexpectedly, the present study showed that four patients (1.5%) believed in spiritual or folk therapy, considering the firm belief of our Islamic society in "Roqia’" or Sheick's spiritual ability for healing. That was also seen in a study conducted in Malaysia, which revealed that the community culture might affect intentions of seeking assistance and set an example that spiritual diagnosis and clergy treatments in East Malaysia may be a reason for medical help-seeking delay [13].

Another study by Shefer et al. surveyed healthcare professionals from four hospitals working with patients attending the emergency department. Healthcare professionals were asked about their practice with patients showing up with mental illness and physical symptoms. The study aimed to explore the challenges in diagnosing physical illness in patients with mental illness. The study concluded that the most common reasons for the misdiagnosis of these patients were complicated presentation and poor communication. Additionally, it ended up recommending that the presence of a psychiatrist with the emergency team is crucial to facilitate the diagnosis of these patients [30].

The above results from Shefer et al. agree with the present work that early referral to a psychiatrist can help better diagnose patients even regarding physical illness. Nevertheless, this study has focused mainly on inpatients admitted to the hospital, whereas our study has focused on patients presenting to outpatient psychiatric clinics. Furthermore, the present work explored the reasons for delaying the first psychiatric visit concerning age.

A study conducted in Ethiopia [31] revealed that individuals aged 31-40 were 10.7 times more likely to seek psychiatric help than those under 20, maybe because older adults have more knowledge about mental illness.

In Singapore, a study also found that younger people are more likely to seek medical help [32]. Nevertheless, there is no specific evidence on the correlation between age group and seeking psychiatric help. On the other hand, some studies [33-34] showed that older adults possess a negative attitude toward psychiatric help, and others [35] totally revealed the opposite. Thus, further investigations need to be done to identify the effect of age, which may depend on knowledge and cultural contexts.

Limitations

The study population is still too small to allow for the establishment of a definitive clinical conclusion, even with the addition of an online questionnaire. Furthermore, neither the general population nor the relevant population is represented by this sample that was chosen. Further extensive research is, therefore, required to fully explore this crucial subject. Due to their hectic schedules or psychological fragility, these patients' lack of excitement for visiting psychiatric clinics constituted another barrier. However, there were no accessible incentives.

Since these studies are prone to biases and confounding variables, prospective studies are a better way to estimate the etiological impact. To the best of my knowledge, this is the first study to look into the reasons why people in Saudi Arabia - especially Taif - delay getting psychiatric assistance. This study may serve as a foundation for developing additional tactics to enhance the psychiatric care provided to our patients.

Recommendations

General population awareness of mental disorders and the available psychiatric services through the health system should be increased. This could be done through national campaigns in clubs, schools, and universities to endorse the importance of early consultation in psychiatric clinics if psychiatric symptoms appear and campaigns against stigma to develop a positive attitude towards seeking psychiatric help. Additionally, early intervention programs should be established as a preventive measure. Finally, further studies are required to represent better the Saudi community, including large samples and multiple centers.

## Conclusions

This study demonstrated that the most common reason for delaying mental health consultation in men and women was the fear of psychotropic medications. Anxiety was the most common psychiatric symptom among patients delaying their first psychiatric consultation regardless of age. These findings serve as a guide for the improvement of mental health services and psychoeducation in Saudi Arabia.
